# Repetitive head injuries in German American football players do not change blood-based biomarker candidates for CTE during a single season

**DOI:** 10.1186/s42466-024-00307-6

**Published:** 2024-02-29

**Authors:** Theres Bastgen, Janis Evers, Christiane Oedekoven, Caroline Weide, Lars Herzog, Nicholas Ashton, Henrik Zetterberg, Kaj Blennow, Alexandra Albus, Natasha Vidovic, Oliver Kraff, Cornelius Deuschl, Richard Dodel, J. Alexander Ross

**Affiliations:** 1 Department of Geriatric Medicine and Center for Translational and Behavioral Neuroscience, University Duisburg-Essen, Essen, Germany; 2 Institute for Health Services Research and Clinical Epidemiology (IVE), Philipps-University, Marburg, Germany; 3 Department of Radiology, University Hospital Essen, Essen, Germany; 4 Department of Psychiatry and Neurochemistry, Institute of Neuroscience and Physiology, Sahlgrenska Academy, University of Gothenburg, Mölndal, Sweden; 5 Institute of Psychiatry, Psychology and Neuroscience Maurice Wohl Institute Clinical Neuroscience Institute, King’s College London, London, UK; 6 NIHR Biomedical Research Centre for Mental Health and Biomedical Research Unit for Dementia at South London and Maudsley NHS Foundation, London, UK; 7 Centre for Age-Related Medicine, Stavanger University Hospital, Stavanger, Norway; 8 Clinical Neurochemistry Laboratory, Sahlgrenska University Hospital, Mölndal, Sweden; 9 Department of Neurodegenerative Disease, UCL Institute of Neurology, Queen Square, London, UK; 10 UK Dementia Research Institute at UCL, London, UK; 11 Hong Kong Center for Neurodegenerative Diseases, Clear Water Bay, Hong Kong, China; 12 Wisconsin Alzheimer’s Disease Research Center, School of Medicine and Public Health, University of Wisconsin, University of Wisconsin-Madison, Madison, WI USA; 13 Therapy Research in Neurogeriatrics, Chair of Geriatric Medicine, University Duisburg-Essen, Virchowstrasse 171, 45174 Essen, Germany; 14 Erwin L. Hahn Institute for MR Imaging, University of Duisburg-Essen, Essen, Germany

**Keywords:** American football, Chronic traumatic encephalopathy, Biomarkers, Traumatic brain injury, Tau protein, Neurofilament light protein

## Abstract

**Background:**

Repetitive traumatic brain injuries in American football players (AFPs) can lead to the neurodegenerative disease chronic traumatic encephalopathy (CTE). Clinical symptoms of CTE range from mood and behavioral changes to cognitive impairment, depression, and suicidality. So far, CTE cannot be diagnosed in vivo and thus specific diagnostic parameters for CTE need to be found, to observe and treat exposed athletes as early as possible. Promising blood-based biomarkers for CTE include total tau (tTau), hyperphosphorylated tau (pTau), neurofilament light protein (NF-L), glial fibrillary acidic protein (GFAP), amyloid-β_40_ (Aβ_40_), amyloid-β_42_ (Aβ_42_) and calcium-binding protein B (S100-B). Previous studies have found elevated levels of these biomarkers in subjects exposed to TBIs, whereas cerebrospinal fluid (CSF) levels of Aβ_40_ and Aβ_42_ were decreased in CTE subjects. Here, we investigated whether young AFPs already exhibit changes of these biomarker candidates during the course of a single active season.

**Methods:**

Blood samples were drawn from n = 18 American Football Players before and after a full season and n = 18 male age-matched control subjects. The plasma titers of tTau, pTau, NF-L, GFAP, Aβ_40_, Aβ_42_ and S100-B were determined. Additionally, Apathy, Depression, and Health status as well as the concussion history and medical care were assessed and analyzed for correlations.

**Results:**

Here we show, that the selected biomarker candidates for CTE do not change significantly during the seven-month period of a single active season of American Football in blood samples of AFPs compared to healthy controls. But interestingly, they exhibit generally elevated pTau titers. Furthermore, we found correlations of depression, quality-of-life, career length, training participation and training continuation with headache after concussion with various titers.

**Conclusion:**

Our data indicates, that changes of CTE marker candidates either occur slowly over several active seasons of American Football or are exclusively found in CSF. Nevertheless, our results underline the importance of a long-term assessment of these biomarker candidates, which might be possible through repeated blood biomarker monitoring in exposed athletes in the future.

**Supplementary Information:**

The online version contains supplementary material available at 10.1186/s42466-024-00307-6.

## Background

American football players (AFPs) suffer repetitive head injuries during their active career, which can result in mild to severe traumatic brain injuries (TBIs). A potential late consequence of repetitive TBIs is the neurodegenerative disease chronic traumatic encephalopathy (CTE) [1], which was first described in boxers as ‘punch drunk syndrome’ [2]. Clinical symptoms of CTE usually appear years to decades after the head injury exposures and range from mood and behavioral changes to cognitive impairment, depression, and suicidality [3]. So far, CTE cannot be diagnosed in vivo. Recently the traumatic encephalopathy syndrome (TES) has been introduced, aiming to describe the unspecific criteria for the clinical presentation of CTE [4]. Yet, specific diagnostic parameters for CTE need to be found, to observe and treat exposed athletes as early as possible. Repetitive TBIs can lead to diffuse axonal injuries (DAIs) and neurostructural changes in the exposed brain [3]. An accumulation of abnormal hyperphosphorylated tau- protein (phospho-Tau, pTau) predominantly occurring in neurons, astroglia and around small blood vessels is the pathognomonic pattern in CTE [5].

Previous studies have found changes in potential biomarkers in the blood (serum and plasma), cerebrospinal fluid (CSF), and saliva of athletes with a history of repetitive TBIs [6–8]. If these biomarkers proof to sensitively identify severity and expanse of neurodegenerative changes in the brain, they could be useful for an early risk evaluation of exposed athletes and potentially predict a clinical outcome [8, 9]. Promising biomarkers include, among others, total tau (tTau), pTau, neurofilament light protein (NF-L), glial fibrillary acidic protein (GFAP), amyloid-β (Aβ_40_, and Aβ_42_) and calcium-binding protein B (S-100B) [9]. Previous studies have found elevated levels of these biomarkers in subjects exposed to TBIs, such as AFPs and boxers [6, 8, 10]. CSF levels of Aβ_40_ and Aβ_42_ were decreased in CTE subjects [10]. Currently, data is available for elder age groups and highly professional players only. The aim of this study was to investigate whether young AFPs may already exhibit pathological changes during the course of a single active season.

## Methods

### Participants and blood sampling

A total of 18 male AFPs (mean age: 24.7 years) were examined pre- (T0) and postseason (T1) and compared with sex-/age-matched controls (CG) (N = 18, mean age: 24.7 years) with no participation in contact sports nor a recent history of concussion. Controls were examined once. Three AFPs dropped out after the T0-examination due to illness or peripheral injury. Blood samples were collected from every subject in 2 × 9 ml serum 2 × 9 ml EDTA monovettes, resulting in a total blood draw of 30 ml from each subject, and were kept at -80 °C until further examination.

### Clinical physical examination and questionnaires

We performed a physical/neurological examination of all participants at baseline/follow-up including assessments of vital parameters.

#### Apathy

Apathy Evaluation Scale (AES). The AES is an instrument assessing the presence and symptoms of apathy. AES is syndrome independent from other diseases that are often associated with apathy, such as depression and anxiety disorders [11, 12]. The AES-C (clinician-rated) was used in our study [13]. A score greater than 34 points indicates clinically relevant apathy on AES-C.

#### Depression

Beck-Depression-Inventory (BDI-II). The BDI-II consists of 21 items which cover the most common symptoms of depression. Each item is rated on a 4-point scale with 0 to 3 points, which correlates with the increasing severity of the symptoms [14, 15].

#### Health status

EuroQuol 5 Dimensions (EQ-5D-5 L). The EQ-5D descriptive system comprises five dimensions [16]: mobility/self-care/usual activities/pain-discomfort/anxiety-depression with 5 levels (no problems/slight/moderate/severe/extreme problems). We computed the EQ-5D-5 L index values utilizing the German value set with an index score of 1.00 corresponding to the best health status possible. In addition, the visual analog scale (VAS) was used [17].

### Questionnaires to record demography, lifestyle, athletic career, concussion history, equipment and medical care

The ‘demographics questionnaire’ included items on age/height/weight/marital status/ethnicity/school years/graduation/vocational training/current employment. Everyday behavior outside of sport and work was documented to measure the level of professionalism. We recorded the consumption of alcohol and tobacco, dietary supplements, sports-related nutrition and other sports beside AF.

Activity: Global Physical Activity Questionnaire (GPAQ). The GPAQ [18] queries the activity behavior in a normal week. It consists of 16 items, of which 15 items relate to physical activity at work, during leisure time and when moving from place to place. The last item relates to physical inactivity.

### Biomarker analysis

EDTA plasma samples were analyzed at the Clinical Neurochemistry Laboratory at Sahlgrenska University Hospital, Sweden. All measurements were performed blinded to the clinical data. Plasma levels of pT231 were determined by an in-house Simoa method developed by the University of Gothenburg, as previously described in detail [19], GFAP, NF-L, and t-Tau levels were measured using the Human Neurology 4-Plex A assay (N4PA) and Aβ_40_ and Aβ_42_ levels were measured with a duplex Simoa immunoassay (Quanterix, Billerica, MA). Levels of S100-B were analyzed in serum samples using the Elecsys® S100 kit on a cobas e601 instrument, following the instructions by the manufacturer.

### Statistical analysis of biomarker titers

Statistically we compared AFPs (T0) with the CG (non-related) and AFPs pre- and postseason data. Data distribution was determined with a Wilcoxon matched-pairs signed rank test. Non-parametric data was further analyzed with the Mann-Whitney-U test (i.e., pTau, GFAP, NF-L, S100-B), whereas parametric data was analyzed with a Student’s *t*-test (i.e., tTau, Aβ_40_, Aβ_42_). In cases of drop-outs a listwise deletion was performed. Significant *p*-values are depicted in the figures by asterisks: * < 0.05.

**Fig. 1:**
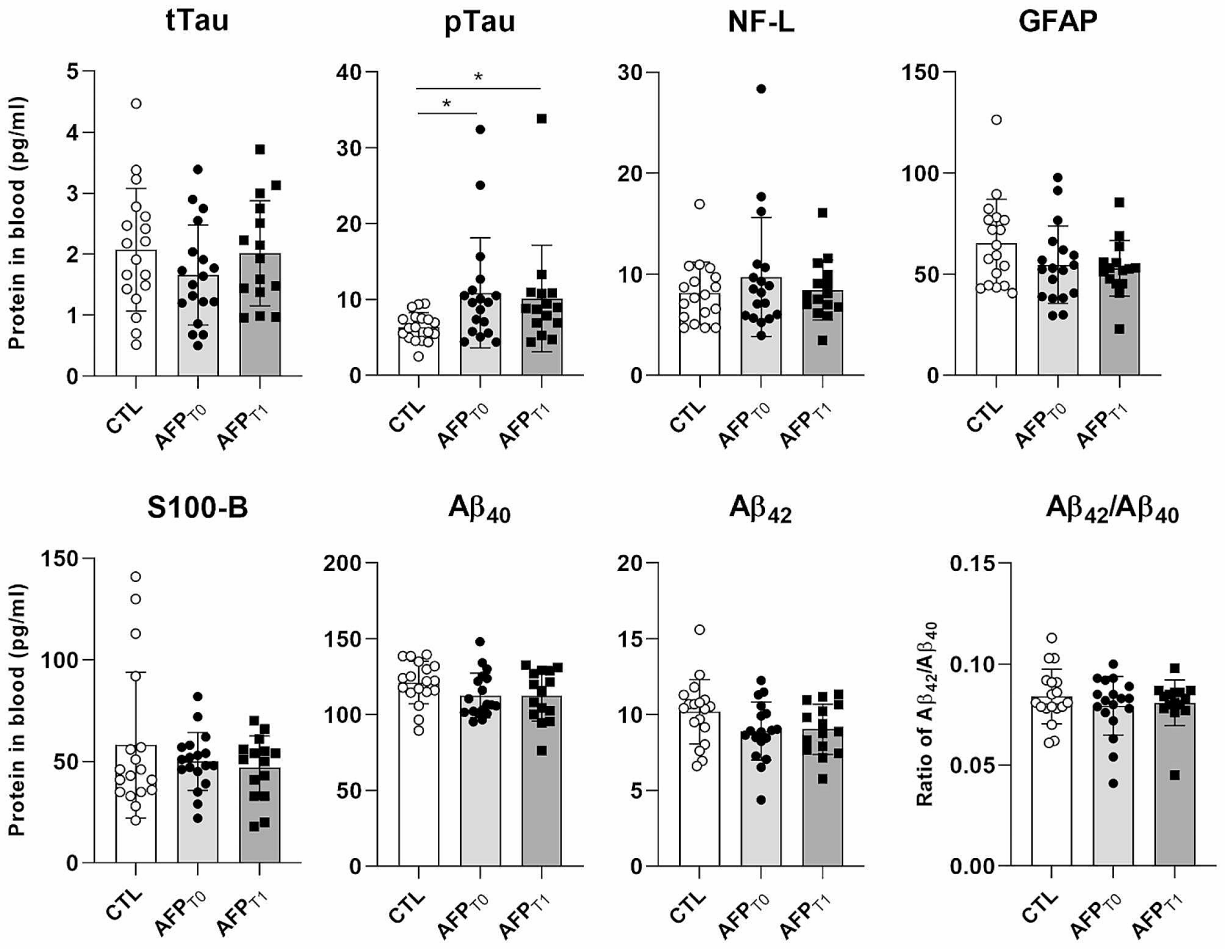
Serum titer of selected chronic traumatic encephalopathy (CTE) biomarker candidates. Blood samples of American football players (AFPs) were taken before (T0) and after (T1) an active season and compared to non-AFPs controls (CG). The serum/plasma was analyzed with ELISA for the titers of total Tau (tTau), phospho-Tau (pTau), neurofilament light protein (NF-L), glial fibrillary acidic protein (GFAP), Amyloid-β 40 (Aβ_40_) and 42 (Aβ_42_) and calcium binding protein B (S100-B). Additionally, the ratio of Aβ_40_ and Aβ_42_ was calculated. The distribution of the data was assessed with the Shapiro-Wilk test. Further statistical analysis was performed with either paired (AFP_T0_ and AFP_T1_) or unpaired t-test (AFP and CG) for normally distributed data or else Wilcoxon matched-pairs signed rank test (AFP_T0_ and AFP_T1_) and Mann Whitney-test (AFP and CG), respectively

### Acquisition and statistical analysis of clinical data

To investigate the strength and direction of the linear relationship between biomarkers and clinical data as well as football-related variables, such as seasons of AF played, participation in practice, number of times played with headaches after a hit and total games played, we performed a Pearson correlation analysis. Clinical parameters for the correlation analyses were the subjects’ depression and apathy status as well as their health-related quality of life. A comprehensive summary of the results can be found in the appendix.

## Results

Blood samples of 18 male AFPs (pre- (T0) and postseason (T1)) and 18 gender-/age-matched CG were taken and selected biomarker titers analyzed. Additionally, clinical data has been collected to investigate possible correlations to the biomarker titers.

### AFPs exhibit increased pTau titers

The titer of pTau was significantly elevated in AFPs (10.87 ± 7.27 pg/ml at T0 and 10.12 ± 7.03 pg/ml at T1) compared to CGs (6.39 ± 1.89 pg/ml), but remained unchanged within the AFP group over time. The mean titer of tTau did increase over time, although not significantly, from 1.66 ± 0.82 pg/ml (T0) to 2.02 ± 0.86 pg/ml (T1), but remained indifferent to CG with 2.08 ± 1.00 pg/ml (Fig. 1).

The titers of Aβ_40_ and Aβ_42_ have previously been reported to be decreased in CSF samples of CTE subjects. In this study we also observed lower titers of Aβ_40_ in AFPs (112.8 ± 14.47 pg/ml (T0) and 112.5 ± 16.76 pg/ml (T1)) compared to CGs (121.1 ± 14.02 pg/ml). Furthermore, we observed the same for Aβ_42_ in AFPs (8.92 ± 1.91 pg/ml (T0) and 9.031.67 pg/ml (T1)) compared to CGs (10.19 ± 2.13 pg/ml). However, the observed differences between the groups as well within the AFP group remained insignificant. The Aβ_42_/Aβ_40_ ratio, which has been shown to be decreased in CTE patients [10], remained unchanged and similar in all groups with 0.08 ± 0.01 in all groups (Fig. 1).

The concentrations of GFAP were lower in AFPs (54.73 ± 19.11 pg/ml (T0) and 52.91 ± 13.76 pg/ml (T1)) compared to CGs (65.37 ± 21.74 pg/ml) and S100-B in AFPs (0.05 ± 0.01 µg/ml (T0) and 0.47 ± 0.02 µg/ml (T1)) compared to CGs (0.06 ± 0.04 µg/ml). Statistical analysis did not indicate that these differences were significant (Fig. 1).

Finally, NF-L concentration was higher in AFPs (9.74 ± 5.88 pg/ml (T0) and 8.45 ± 2.97 pg/ml (T1)) compared to CGs (8.13 ± 3.08 pg/ml). All differences between the AFP group and CG group, as well as between T0 and T1 within the AFPs were not significant (Fig. 1).

### Biomarker correlations to clinical data

To evaluate possible correlations between biomarker titers and clinical data, we performed correlation analyses. Significant results were then assessed regarding their mean threshold value (depression yes/no) or median splits with the separation value (x).

We found a negative correlation between the subjects’ depression status and Aß_42_ titers (Table 1, S1). Subjects with a mild or moderate depression (BDI II ≥ 9 points) have lower Aß_42_ titers in both T0 (r = -0.578; n = 3; mean: 6.71; 95%-CI [1.45;11.98]) and T1 (r = -0.601; n = 3; mean: 6.87; 95%-CI [4.40;9.33]) compared to subjects with no depression (BDI II < 9 points; T0: n = 15; mean: 9.36; 95%-CI [8.47;10.24]; T1: n = 12; mean: 9.57; 95%-CI [8.47;10.24]).

Furthermore, we found a positive correlation between the subjects’ depression status and NF-L titers (T1) (Table 1, S1). Subjects with a mild or moderate depression (BDI II ≥ 9 points) have higher NF-L titers (r = 0.519; n = 3; mean: 10.72; 95%-CI [-0.84;22.28]) compared to subjects with no depression (BDI II < 9 points; n = 12; mean: 7.89; 95%-CI [6.39;9.38]).

Health related quality of life, measured with the Eq. 5D-5 L (x = 0.974) was positively correlated with GFAP titers (T0) (Table 1, S2). The lower the subjects’ health related quality of life (Eq. 5D-5 L < 0.974), the lower was the GFAP titer Eq. 5D-5 L (r = 0.489; n = 15; mean: 52.79; 95%-CI [43.32;62.26]), compared to subjects with a higher health related quality of life (Eq. 5D-5 L ≥ 0.974; n = 3; mean: 64.40; 95%-CI [-9.67;138.48]).

Further, we found a positive correlation between the subjects’ length of their AF careers (x = 6) and Total-Tau titers (T1) (Table 1, S3). Subjects, that have played for 6 or more seasons had higher Total-Tau titers (r = 0.645; n = 9; mean: 2.71; 95%-CI [2.04;3.39]), compared to subjects, that have played for less than 6 seasons (n = 9; mean: 1.55; 95%-CI [1.04;2.06]).

A negative correlation regarding the amount of trainings’ participation (x = 0.85) and Aß_42_ titers (T1) were also found (Table 1). Subjects with a trainings’ participation of 85% or more within the last season had lower Aß_42_ titers (r = -0.554; n = 8; mean: 8.51; 95%-CI [7.17;9.86]), compared to subjects with a training participation of less than 85% (n = 7; mean: 9.63; 95%-CI [8.12;11.14]).

Subjects, that continued playing with headaches (Table 1, S4) after an occurred head injury (x = 0.50) in 50% of the times or more (T1), had higher S100-B titers (r = 0.592; n = 9; mean: 0.05; 95%-CI [0.04;0.06]), compared to subjects, that continued playing in less than 50% of the times (n = 6; mean: 0.04; 95%-CI [0.03;0.05]).

No significant clinical correlation could be found regarding pTau titers.

**Table 1:** Overview of clinical data correlation with biomarker titers

Parameter	Value	Biomarker	Correlation	Comment
Depression	+	Aβ_42_	↓	
Depression	+	NF-L	↑	
Health-related QoL	↑	GFAP	↓	
Length of career	↑	tTau	↑	When career ≥ 6 season of length
Training participation	↑	Aβ_42_	↓	When participated ≥ 80% of training sessions
Continuation with Headache	↑	S100-B	↑	When continued after head injury with subsequent headache in ≥ 50% cases

The symbols indicate values as: + = diagnosed; ↑ = elevated; ↓ = decreased

## Discussion

In this study, we found that selected biomarker candidates which are investigated in the context of concussion and CTE do not change over the period of one active season in blood samples of AFPs. Also, most do not differ from CGs with the exception, that pTau was elevated in AFPs compared to CGs. However, this value did not differ after a season within the same group.

Interestingly, NF-L CSF titer has previously been shown to be significantly elevated after an active season in AFPs [7]. One study also found increasing levels of serum NF-L in American football athletes over the course of a season, specifically in starters versus non-starters [7]. Although we did not detect significant changes in the titer over the course of a season or between AFPs and CGs, we recognized that NF-L is positively correlated with the occurrence of depression.

In another study, conducted on a cohort of former, CTE-symptomatic AFPs, the tTau plasma titer was correlated to the estimated count of head injuries, although there were no significant differences to the CG group [20]. Additionally, in further experiments on the same cohort, tau-positive exosomes in plasma were found to be significantly elevated in the group of former AFPs [21]. This was reflected in our data by the elevated titer of pTau in AFPs compared to CG, although, this did not change during the active season. Interestingly, we found that tTau is correlated to the length of the AF career when dividing the career length into 5 seasons or less and 6 seasons or more, indicating a long-term increase of tTau, which still has been undetectable in comparison to CGs. Recently, MTBR-tau has been described as a specific biomarker for tau tangle pathology [22]. Until now, MTBR-tau is detectably in CSF only and was therefore not investigated here. In summary, tTau should be investigated in further studies and observed in long-term and, if applicable in the future, MTBR-tau should be included.

S100-B and GFAP are astrocyte-associated proteins, which have been shown to be slightly elevated in CSF of boxers [6, 8]. Similar to the NF-L, this change might be exclusive to the CSF or at least, is too small to be recognized in the plasma. Still, S100-B was positively correlated to individuals who continued to play after suffering a head injury accompanied with subsequent headache in at least 50% of the cases. Although this seems to fit to the known titer changes of boxers, we could not find the same correlation for GFAP. More contrary, we found it to be positively correlated to the Health-based quality of life.

The titers of Aβ_40_ and Aβ_42_ were, although not significantly, lower in AFPs at both timepoints and therefore should be investigated over a longer period to investigate, if they change during prolonged repetitive TBIs. Importantly, we found correlations to the depression status and the frequency of training participation. AFPs with depression and, independently of that, those who participated in at least 80% of the training sessions had lower Aβ_42_ titers, correlated to Alzheimer’s disease.

## Conclusion

Contrary to prior research [23], our study suggests that the selected biomarkers for CTE do not change significantly during a single active season of AF in AFPs. However, when observed consistently over an extended period, they might indicate the development of CTE. Thus, a longitudinal study with a similar setting but increased number of participants would be reasonable. Furthermore, additional markers (e.g., ApoE) and intracellular parameter should be investigated in peripheral mononuclear cells. The analysis of microstructural changes in the brains of the investigated cohort would be also an important tessellation. The results, however, would go beyond the scope of this article and will be presented in an upcoming article. Finally, our study showed that clinical and psychological data might be a valuable addition to predict changes in the biomarker titers. Notably, our results indicated correlations between psychological factors and protein titers, although they do not differ between CTL and AFPs themselves. If such correlations can reliably be found, these needs to be investigated in a study as outlined before.

Summarized, our findings indicate possible CTE markers observable during a professional sports career and facilitate early diagnosis and treatment of subjects affected by CTE. However, due to the small sample size, all findings should be interpreted with caution and can only be considered indicative. Finally, it should be noted that our investigations focus specifically on CTE following acute injury and may not necessarily extend to tauopathies in general.

### Electronic supplementary material

Below is the link to the electronic supplementary material.

Supplementary Material 1

## Data Availability

All datasets generated and/or analyzed during this study are available from the corresponding author on reasonable request.

## Abbreviations

Aβ_40_: Amyloid-β_40_
Aβ_42_: Amyloid-β_42_
AES: Apathy Evaluation Scale
AFP: American football player
BDI-II: Beck-Depression-Inventory
CTE: Chronic traumatic encephalopathy
CG: Sex-/age-matched controls
CSF: Cerebrospinal fluid
DAI: Diffuse axonal injury
EQ-5D-5L: EuroQuol 5 Dimensions
GFAP: Glial fibrillary acidic protein
GPAQ: Global Physical Activity Questionnaire
N4PA: Neurology 4-Plex A assay
NF-L: Neurofilament light protein
S100-B: Calcium-binding protein B
pTau: Hyperphosphorylated tau
TBI: Traumatic brain injury
TES: Traumatic encephalopathy syndrome
tTau: Total Tau
VAS: Visual analog scale
