# Supplementary material for: Repetitive head injuries in German American football players do not change blood-based biomarker candidates for CTE during a single season
Source: Neurol Res Pract. 2024 Feb 29;6:13. doi: 10.1186/s42466-024-00307-6 (PMC10903054; doi:10.1186/s42466-024-00307-6)
Supplement: Supplementary file 1 — Supplementary Material 1 [file 42466_2024_307_MOESM1_ESM.docx]

**Supplemental Table 1: Correlation between biomarker titer and American football players’ (AFPs) depression status.** Blood samples and clinical questionnaires were taken before (T0) and after (T1) an active season. The AFPs’ depression status was assessed with the Beck-Depressions-Inventar II (BDI II). A score lower than 9 points indicates no depression. A score of 9 or more points indicates a depression. Correlation analyses were then performed. Significant results were then assessed regarding their mean threshold value (depression yes/no). Amyloid beta 42 (Aβ_42_) in µg/ml, neurofilament light protein (NF-L) in pg/ml, Confidence interval (CI). Aβ_42_ T0 r=-0.578. Aβ_42_ T1 r=-0.601. NF-L T1 r=0.519.

| Biomarker | | BDI II points | N | Mean | 95%-CI low | 95%-CI high |
| --- | --- | --- | --- | --- | --- | --- |
| Aβ_42_ | T0 | < 9 | 15 | 9.36 | 8.47 | 10.24 |
|  |  | ≥ 9 | 3 | 6.71 | 1.45 | 11.98 |
|  | T1 | < 9 | 12 | 9.57 | 8.73 | 10.42 |
|  |  | ≥ 9 | 3 | 6.87 | 4.40 | 9.33 |
| NF-L | T1 | < 9 | 12 | 7.89 | 6.39 | 9.38 |
|  |  | ≥ 9 | 3 | 10.72 | -0.84 | 22.28 |

**Supplemental Table 2: Correlation between biomarker titer and amount of American football (AF) played regarding training participation or seasons played.** Blood samples and sociodemographic questionnaires were taken before (T0) and after (T1) an active season. Correlation analyses were then performed. Significant results were then assessed regarding their median splits with the separation value (x). Amyloid beta 42 (Aβ_42_) in µg/ml, total-Tau (tTau) in µg/ml, confidence interval (CI), Pearson correlation (r). *Training participation in %, x=0.85, r=-0.554 (Aβ_42_). **Seasons played, x=6, r=0.645 (tTau).

| Biomarkers | | Amount of AF played | N | Mean | 95%-CI low | 95%-CI high |
| --- | --- | --- | --- | --- | --- | --- |
| Aβ_42_ | T1 | < 85 %* | 7 | 9.63 | 8.12 | 11.14 |
|  |  | ≥ 85 %* | 8 | 8.51 | 7.17 | 9.86 |
| tTau | T1 | < 6** | 9 | 1.55 | 1.04 | 2.06 |
|  |  | ≥ 6** | 9 | 2.71 | 2.04 | 3.39 |

**Supplemental Table 3: Correlation between biomarker titer and American football players’ (AFPs) health related quality of life (HRQL).** Blood samples and clinical questionnaires were taken before (T0) and after (T1) an active season. HRQL was assessed with the European Quality of Life 5 Dimensions 5 Level (EQ-5D-5L) questionnaire. Correlation analyses were then performed. Significant results were then assessed regarding their median splits with the separation value (x). Glial fibrillary acidic protein (GFAP) pg/ml, confidence interval (CI), Pearson correlation (r). X=0.974, r=0.489.

| Biomarkers | | HRQL | N | Mean | 95%-CI low | 95%-CI high |
| --- | --- | --- | --- | --- | --- | --- |
| GFAP | T0 | < 0.974 | 15 | 52.79 | 43.32 | 62.26 |
|  |  | ≥ 0.974 | 3 | 64.40 | -9.67 | 138.48 |

**Supplemental Table 4: Correlation between biomarker titer and frequency that American football players (AFPs) continued playing with headaches after a head injury occurred (in %).** Blood samples and sociodemographic questionnaires were taken before (T0) and after (T1) an active season. Correlation analyses were then performed. Significant results were then assessed regarding their median splits with the separation value (x). Calcium binding protein B (S100B) in µg/ml, confidence interval (CI), Pearson correlation (r). X=0.50, r= 0.592.

| Biomarkers | | Played with headaches | N | Mean | 95%-CI low | 95%-CI high |
| --- | --- | --- | --- | --- | --- | --- |
| S100B | T1 | < 50 % | 6 | 0.04 | 0.03 | 0.05 |
|  |  | ≥ 50 % | 9 | 0.05 | 0.04 | 0.06 |
